# Cost-effectiveness of Novel Macrophage-Regulating Treatment for Wound Healing in Patients With Diabetic Foot Ulcers From the Taiwan Health Care Sector Perspective

**DOI:** 10.1001/jamanetworkopen.2022.50639

**Published:** 2023-01-12

**Authors:** Hsuan-Yu Su, Chen-Yi Yang, Huang-Tz Ou, Shyi-Gen Chen, Jui-Ching Chen, Hui-Ju Ho, Shihchen Kuo

**Affiliations:** 1 Institute of Clinical Pharmacy and Pharmaceutical Sciences, College of Medicine, National Cheng Kung University, Tainan, Taiwan; 2 Department of Pharmacy, College of Medicine, National Cheng Kung University, Tainan, Taiwan; 3 Department of Medical Science, Oneness Biotech Co, Ltd, Taipei, Taiwan; 4 Department of Surgery, Tri-Service General Hospital, Taipei, Taiwan; 5 Department of Clinical Research, Oneness Biotech Co, Ltd, Taipei, Taiwan; 6 Division of Metabolism, Endocrinology & Diabetes, Department of Internal Medicine, University of Michigan Medical School, Ann Arbor

## Abstract

**Importance:**

Diabetic foot ulcers (DFUs) and subsequent amputation incur enormous health and economic burdens to patients, health care systems, and societies. As a novel macrophage-regulating drug, ON101 is a breakthrough treatment for DFUs, which demonstrated significant complete wound healing effects in a phase 3 randomized clinical trial, but its economic value remains unknown.

**Objective:**

To assess the cost-effectiveness of an ON101 cream added on to general wound care (GWC; ie, conventional treatments for DFUs, which comprised initial and regular foot examinations, ulcer management, comorbidity control, patient education, and multidisciplinary care) vs GWC alone for DFUs from the Taiwan health care sector perspective.

**Design, Setting, and Participants:**

This economic evaluation used a hypothetical cohort of patients with diabetes, with characteristics mirroring those of the participants in the ON101 trial. A Markov state-transition simulation model was constructed to estimate costs and health outcomes associated with the ON101 with GWC and GWC alone strategies over a 5-year time horizon, discounting costs and effectiveness at 3% annually. Costs were in 2021 US dollars. Data were sourced from the ON101 trial and supplemented from published literature. Deterministic and probabilistic sensitivity analyses were performed to assess the uncertainty of input parameters and study generalizability. The analysis was designed and conducted from September 1, 2020, to January 31, 2022.

**Exposures:**

ON101 with GWC vs GWC alone.

**Main Outcomes and Measures:**

DFU-related complications, costs, quality-adjusted life-years (QALYs), and incremental cost-effectiveness ratio.

**Results:**

Patients in the hypothetical cohort had a mean age of 57 years and an uninfected DFU of 1 to 25 cm^2^ that was present for 4 or more weeks with a Wagner grade of 1 or 2. Over 5 years, the ON101 with GWC group vs the GWC alone group experienced more healing events, stayed for a longer time in the healing state, and had fewer infected DFUs, gangrene, and amputations (eg, 2787 additional healing events and 2766 fewer infected DFU, 72 fewer amputation, and 7 fewer gangrene events in the ON101 with GWC group vs GWC alone group). The ON101 with GWC strategy vs GWC alone yielded an additional 0.038 QALYs at an incremental cost of $571, resulting in $14 922/QALY gained. Economic results were most sensitive to healing efficacy, drug cost, and health utility of the healing state. Cost-saving results were observed in patient subgroups with poor glycemic control, larger ulcer sizes, longer ulcer durations, and current smoking. The ON101 with GWC strategy was considered cost-effective in 60% to 82% of model iterations against willingness-to-pay thresholds of $32 787/QALY gained to $98 361/QALY gained.

**Conclusions and Relevance:**

In this economic evaluation study using a simulated patient cohort, the ON101 with GWC strategy represented good value compared with GWC alone for patients with DFUs from the Taiwan health care sector perspective and may be prioritized for those with high risks for disease progression of DFUs.

## Key Points

**Question:**

Is adding a novel macrophage-regulating drug, ON101 cream, to general wound care (GWC) cost-effective compared with GWC alone for treating diabetic foot ulcers (DFUs) from a health care sector perspective?

**Findings:**

In this economic evaluation, the ON101 with GWC strategy vs GWC alone strategy gained more wound healing events, averted more DFU-related complications, and cost $14 922/quality-adjusted life-year gained.

**Meaning:**

In this study, ON101-enhanced GWC was cost-effective vs GWC alone at a willingness-to-pay threshold of $32 787/quality-adjusted life-year from the Taiwan health care sector perspective and may be considered in future standard wound care.

## Teaser

This economic evaluation assesses the cost-effectiveness of an ON101 cream added on to general wound care vs general wound care alone for diabetic foot ulcers (DFUs) from the Taiwan health care sector perspective.

## Introduction

Diabetic foot ulcers (DFUs) are among the most serious complications among patients with diabetes. The estimated lifetime incidence of a DFU is 19% to 34%, and roughly 40% to 65% of patients with diabetes undergo recurrence within 1 to 5 years after ulcer healing.^1^ DFUs are prevalent: 6.3% globally and 5.5% in Asia.^2^ Patients with DFUs are at increased risks for recurrence, infection, gangrene, and ultimately amputation, leading to reduced quality of life (QoL) and substantial economic burden.^1,3,4^ In the United States, the per-person incremental annual health care costs for DFUs were $11 710 for Medicare and $16 883 for private insurers.^5^ In England, the annual health care costs for DFUs were £837 million.^6^ In Taiwan, the per-person annual health care costs were $4702 in the year when a DFU occurred and $3284 in the following years.^7^

To ameliorate health and economic burdens of DFUs, the primary treatment goals include accelerating wound healing, remaining longer in the healing state (ie, a healed ulcer), and preventing gangrene and amputation.^8^ Existing treatments for DFUs, however, are limited and most are supportive care using medical equipment rather than pharmacological therapies.^9^ Unfortunately, with currently available treatments, only 35% of DFUs in general healed within 12 months, the mean time to healing was 4.4 months, and 17% of patients with DFUs underwent amputation.^10^ Identifying effective and cost-effective interventions for DFUs is thus of great urgency.

ON101 is a first-in-class novel drug to treat DFUs through regulating M1/M2 macrophages.^11^ ON101 is a breakthrough treatment for DFUs. A phase 3 randomized clinical trial lasting 28 weeks found ON101 to have superior complete wound healing efficacy compared with an absorbent dressing, and such effects could be sustained in patients with poor prognosis of DFUs.^12^ Also, ON101 is formulated in a cream for the convenience of use at home and in outpatient settings to reduce health care burden.

Although the promising efficacy and safety of ON101 were observed in a short-run trial, it remains unknown whether the use of ON101 could provide longer-term economic benefits of treating DFUs. We conducted a computer simulation model to assess the cost-effectiveness of ON101 added on to general wound care (GWC) vs GWC alone for DFUs from the Taiwan health care sector perspective.

## Methods

This economic evaluation was approved by the institutional review board of National Cheng Kung University Hospital before its commencement. Informed consent was waived because this study did not include any human participants. Economic analyses complied with the International Society for Pharmacoeconomics and Outcomes Research (ISPOR) recommendations and were reported in compliance with the Consolidated Health Economic Evaluation Reporting Standards 2022 (CHEERS) checklist.^13^

### Study Model and Simulation

To estimate the cost-effectiveness of ON101 with GWC, a Markov state-transition model, which simulated the chronic and recurrent events in the disease progression of a DFU, was applied to consider a hypothetical cohort of patients with diabetes with a mean age of 57 years who had uninfected DFUs sized 1 to 25 cm^2^, present for at least 4 weeks, and with a Wagner grade of 1 or 2, which was consistent with the characteristics of the participants in the ON101 trial.^12^ Briefly, this multicenter, phase 3 randomized clinical trial was conducted in 21 clinical sites across 3 countries: the United States, China, and Taiwan. Participants with type 1 or 2 diabetes aged 20 to 80 years, with a baseline hemoglobin A_1c_ (HbA_1c_) level of less than 12% (to convert to proportion of total hemoglobin, multiply by 0.01), and with debrided DFUs of 1 to 25 cm^2^ without active infection, present for at least 4 weeks, and with Wanger grade of 1 or 2 were randomized 1:1 to receive ON101 or a control absorbent dressing for 16 weeks of treatment with a 12-week follow-up.^12^ According to the latest treatment guidelines for DFUs, the GWC included initial and regular foot examination, ulcer management, comorbidity control, patient education, and multidisciplinary care.^9,14^

**Figure 1.**
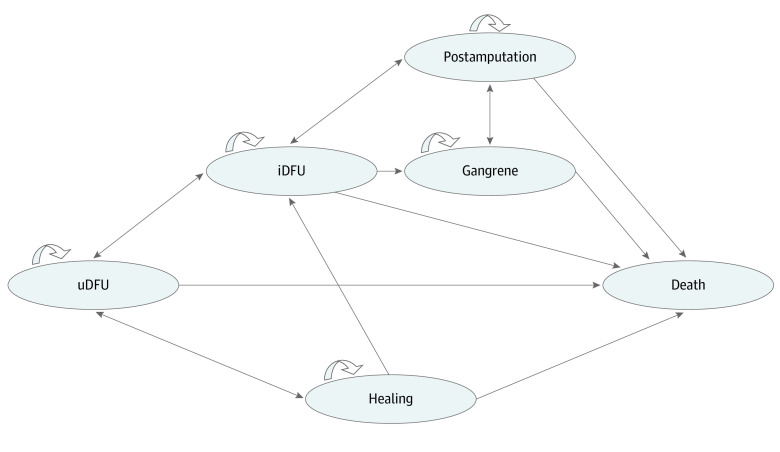
Six Health States in the Markov State-Transition Model iDFU indicates infected diabetic foot ulcer; uDFU, uninfected diabetic foot ulcer.

The Markov model in this study was adapted from existing validated decision-analytic models^15,16^ and examined by clinical experts and experienced modelers (ie, face validation). In particular, the health state of gangrene was retained in the model considering its clinical significance and high prevalence rate. The model simulated DFU progression through 6 mutually exclusive health states (Figure 1). ON101 treatment is indicated only for patients with uninfected or infected ulcers. As commonly applied in previous cost-effectiveness studies,^16,17,18,19^ a 1-month cycle length was used to capture DFU progression, and a 5-year time horizon was used for model simulation in the base-case analysis. Probabilistic sensitivity analyses (PSAs) with 10 000 model iterations were performed where all model input parameters varied simultaneously in the given plausible statistical distributions (Table 1).^7,12,16,18,20,21,22^

**Table 1.** Transition Probabilities, Health Utilities, Costs, and Odds Ratio for Model Inputs in 1-Month Cycle Length

Parameter	Base-case value (range)	Distribution	Source
Transition probability			
uDFU to healing	0.259 (0.1295-0.3885)	Beta	Wu et al,^20^ 2018
uDFU to iDFU	0.068 (0.034-0.102)		Wu et al,^20^ 2018
uDFU to death	0.001 (0.0005-0.0015)		Wu et al,^20^ 2018
iDFU to iDFU	0.084 (0.042-0.126)		Cheng et al,^18^ 2017
iDFU to postamputation	0.016 (0.008-0.024)		Cheng et al,^18^ 2017
iDFU to gangrene	0.036 (0.018-0.054)		Redekop et al,^21^ 2004
iDFU to death	0.001 (0.0005-0.0015)		Cheng et al,^18^ 2017; Redekop et al,^21^ 2004
Healing to progression	0.038 (0.019-0.057)		Ragnarson et al,^16^ 2001; Cheng et al,^18^ 2017
Healing to iDFU	0.211 (0.1055-0.3165)		Ragnarson et al,^16^ 2001; Cheng et al,^18^ 2017
Healing to death	0.001 (0.0005-0.0015)		Ragnarson et al,^16^ 2001; Cheng et al,^18^ 2017
Postamputation to iDFU	0.007 (0.0035-0.0105)		Ragnarson et al,^16^ 2001; Cheng et al,^18^ 2017
Postamputation to gangrene	0.43 (0.215-0.645)		Ragnarson et al,^16^ 2001; Cheng et al,^18^ 2017
Postamputation to death	0.003 (0.0015-0.0045)		Ragnarson et al,^16^ 2001
Gangrene to postamputation	0.313 (0.1565-0.4695)		Ragnarson et al,^16^ 2001
Gangrene to death	0.01 (0.005-0.015)		Ragnarson et al,^16^ 2001; Cheng et al,^18^ 2017
Health utility			
Healing	0.84 (0.756-0.924)	Uniform	Kuo et al,^22^ 2021
uDFU	0.75 (0.675-0.825)		Redekop et al,^21^ 2004
iDFU	0.7 (0.63-0.77)		Redekop et al,^21^ 2004
Gangrene	0.69 (0.621-0.759)		Kuo et al,^22^ 2021
Postamputation	0.59 (0.531-0.649)		Kuo et al,^22^ 2021
Cost in 2021 US $			
Initial DFU	161 (80-241)	Triangular	Chen et al,^7^ 2020; Cheng et al,^18^ 2017
Healing	9 (4-13)		Chen et al,^7^ 2020; Cheng et al,^18^ 2017
uDFU	97 (49-146)		Chen et al,^7^ 2020; Cheng et al,^18^ 2017
iDFU	160 (80-240)		Chen et al,^7^ 2020; Cheng et al,^18^ 2017
Gangrene	231 (116-347)		Chen et al,^7^ 2020; Cheng et al,^18^ 2017
Postamputation	504 (252-756)		Chen et al,^7^ 2020; Cheng et al,^18^ 2017
Hospitalization for amputation^a^	7552 (3776-11 328)		Chen et al,^7^ 2020; Cheng et al,^18^ 2017
Hospitalization for infection^a^	8847 (4423-13 270)		Chen et al,^7^ 2020; Cheng et al,^18^ 2017
Hospitalization for postamputation infection^a^	13 583 (6791-20 374)		Chen et al,^7^ 2020; Cheng et al,^18^ 2017
Hospitalization for postamputation gangrene^a^	13 583 (6791-20 374)		Chen et al,^7^ 2020; Cheng et al,^18^ 2017
Monthly drug cost^b^	608 (304-912)		Huang et al,^12^ 2021
Odds ratio			
Healing effect size of ON101 with GWC vs GWC alone	2.30 (1.15-3.45)	Log normal	Huang et al,^12^ 2021

Abbreviations: DFU, diabetic foot ulcer; GWC, general wound care; iDFU, infected diabetic foot ulcer; uDFU, uninfected diabetic foot ulcer.

^a^ Event costs were for the transition from 1 health state to another.

^b^ Data were estimated from the efficacy analysis report provided by the pharmaceutical company.

### Model Inputs: Transition Probabilities, Health Utilities, and Costs

Transition probabilities reflecting risks of progression between health states were derived from published literature (Table 1). Transition probabilities between any health states were assumed the same for both study groups except for that from uninfected DFU to healing, which reflected the better healing efficacy of ON101 and was factored by the odds ratio (OR) from the ON101 trial to achieve healing.^12^ Briefly, in the trial, there were statistically significant different wound closure and healing rates in the ON101 group, but the ulcer reduction outcomes, including changes in ulcer area from baseline and rate of 50% reduction in wound, were not statistically significantly different between treatment groups.^12^

Quality-adjusted life-years (QALYs) were the primary effectiveness outcome and estimated by multiplying health utilities with the number of years spent in each health state. Health utilities were obtained from the study by Kuo et al^22^ using the population-based data among patients with diabetes in Taiwan, except for the utilities of uninfected and infected DFUs which were adopted from the study by Redekop et al.^21^ Conservatively, all health utilities were assumed the same for both study groups (Table 1).

Monthly health care costs were estimated. Costs were classified as a one-time event or hospitalization costs, which occurred when any health states changed to health states of infection, gangrene, or amputation, and state costs, which represented medical costs incurred in every modeling cycle (Table 1). The total health care costs for each patient represented the sum of the total medical costs associated with health states and the cost of ON101. A 2-stage cost transformation process was applied to adapt the cost data derived from the study by Cheng et al,^18^ which was conducted in Australia, to this study setting through the study by Chen et al,^7^ which was conducted in Taiwan (eFigure 1 in Supplement 1). Briefly, in the first step (eFigure 1a in Supplement 1), costs from the 2 studies^7,18^ were converted into the same currency (US dollars) and standardized to year 2021 values using Taiwan’s medical Consumer Price Index.^23^ Next, cost ratios for transformation (ratios 1 and 2 in eFigure 1b in Supplement 1) were calculated by dividing the Taiwan’s cost estimates by the Australian estimates for the health states that were available in both countries’ data.

### Statistical Analysis

#### Cost-effectiveness Analysis

In the base-case analysis, total health care costs, life expectancy, and QALYs per patient in both groups were simulated over 5 years from the Taiwan health care sector perspective. The cumulative probabilities of patients with healing, uninfected DFU, infected DFU, and postamputation as well as the mean time of staying in the healing health state were computed for each study group. The numbers of healing events and DFU-related complications were estimated through 10 000 times of microsimulation.

The incremental cost-effectiveness ratio (ICER) of ON101 with GWC vs GWC alone was the incremental total health care costs divided by the incremental total QALYs. Future costs and health outcomes were discounted at 3% yearly.^24^ Per recommendations,^24,25,26^ 1 and 3 times the per-capita gross domestic product of Taiwan in 2021 was used as the willingness-to-pay (WTP) threshold to determine whether ON101 with GWC vs GWC alone was highly cost-effective (ICER, ≤$32 787) or cost-effective (ICER, $32 788 to ≤$98 361).

All study analyses were performed using TreeAge Pro 2020 (TreeAge Software, LLC). The impact inventory for the components considered in economic analyses is provided in eTable 1 in Supplement 1.

#### Sensitivity Analyses

A series of sensitivity analyses were performed to assess the impact of parameter uncertainties on the base-case ICER and examine the generalizability of study results. Deterministic sensitivity analyses (DSAs) were conducted in which all input values were varied within a predefined range (ie, ±50% of the base-case value for probabilities, ORs, and costs; ±10% of the base-case value for health utilities) (Table 1). The influential parameters whose variations yielded a base-case ICER change greater than 40% were illustrated in a tornado diagram. The simulation time horizon was restricted to 1 year to examine the short-term cost-effectiveness of ON101 with GWC. Given that both the cost and healing efficacy of ON101 were the factors of clinical interest, a 2-way sensitivity analysis was conducted to examine their interplay on economic results. A break-even analysis was conducted to determine the marginal cost of ON101 under the WTP thresholds.

#### Subgroup Analyses

Subgroup analyses were conducted according to baseline HbA_1c_ levels, ulcer sizes, ulcer durations, and smoking status to explore possible heterogeneity in economic analyses. All model input parameters in subgroup analyses were the same as those in the base-case analysis except for the relative treatment effect sizes for ON101 with GWC vs GWC alone (ie, the OR values) on wound healing, which were specific to patient subgroups derived from the ON101 trial.^12^

## Results

### Base-Case Analysis

The hypothetical cohort of patients with diabetes had a mean age of 57 years and an uninfected DFU of 1 to 25 cm^2^ that was present for 4 or more weeks with a Wagner grade of 1 or 2. Over 5 years, for the ON101 with GWC group, the cumulative probabilities of patients with healing, uninfected DFU, infected DFU, and postamputation health states were 85.6%, 6.5%, 1.0%, and 1.0%, respectively, and the mean time staying in the healing health state was 52.6 (95% CI, 52.4-52.8) months; for those in the GWC alone group, the cumulative probabilities were 79.7%, 11.6%, 1.3%, and 1.4%, respectively, and the mean time staying in the healing health state was 47.4 (95% CI, 47.2-47.6) months (eTables 2 and 3 in Supplement 1). In 10 000 microsimulations, 2787 additional healing events were gained, and 2766 infected DFU, 72 amputation, and 7 gangrene events were averted from using ON101 with GWC vs GWC alone (eTable 3 in Supplement 1).

The ON101 with GWC vs GWC alone yielded a marginal gain of life expectancy and a gain of 0.038 QALYs at an additional cost of $571, resulting in an ICER of $14 922/QALY gained (Table 2). This was much lower than the WTP threshold of $32 787, suggesting that ON101 with GWC vs GWC alone was considered highly cost-effective.

**Table 2.** Base-Case Analysis Results Over a 5-Year Model Simulation

Treatment group	Total cost, 2021 $	Incremental cost, 2021 $	Life expectancy, y	QALY	Incremental QALY	ICER, 2021 $/QALY gained	Probability of cost-effectiveness, %^a^
GWC alone	9210	NA	4.5128	3.7019	NA	NA	NA
ON101 with GWC	9781	571	4.5135	3.7402	0.0383	14 922	82

Abbreviations: GWC, general wound care; ICER, incremental cost-effectiveness ratio; NA, not applicable; QALY, quality-adjusted life-year.

^a^ The probability was against the willingness-to-pay threshold of $98 361/QALY gained.

### Sensitivity Analyses

**Figure 2.**
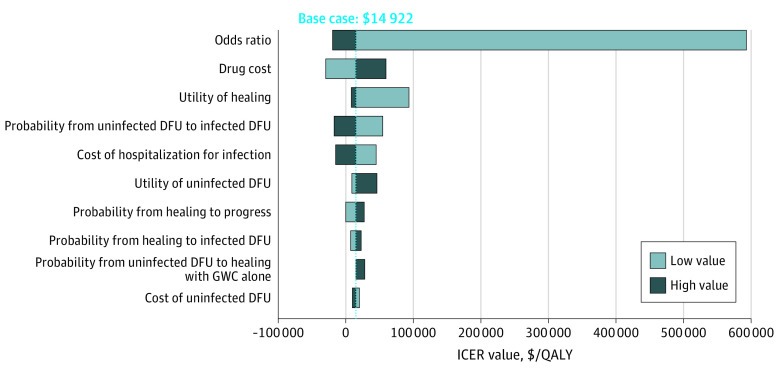
Tornado Diagram for Deterministic Sensitivity Analysis Results The figure shows the incremental cost-effectiveness ratio (ICER) of ON101 with general wound care (GWC) vs GWC alone for different model input parameters. The influential parameters whose variations yielded a base-case ICER change greater than 40% are illustrated in the figure. DFU indicates diabetic foot ulcer; QALY, quality-adjusted life-year.

**Figure 3.**
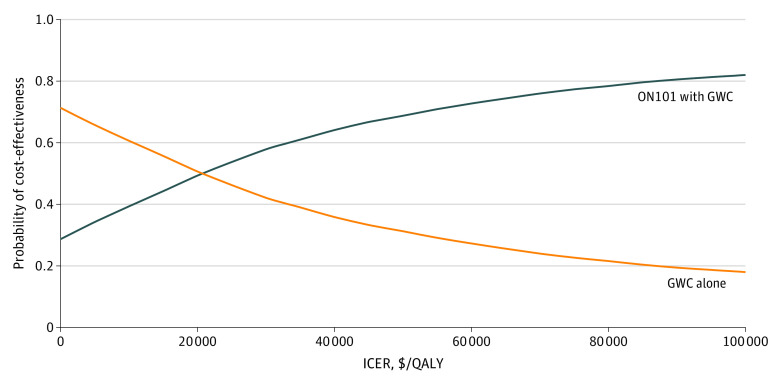
Cost-effectiveness Acceptability Curve The figure shows the probabilistic sensitivity analysis using Monte Carlo simulation with 10 000 iterations, in which the input parameters varied simultaneously in the plausible statistical distributions that reflected the uncertainty of the cost-effectiveness for ON101 with general wound care (GWC) vs GWC alone at different willingness to pay thresholds. ON101 with GWC had a 28.7%, 59.7%, and 81.8% probability of being cost-effective against the willingness to pay thresholds of $0, $32 787, and $98 361 per quality-adjusted life-year (QALY) gained, respectively. ICER indicates incremental cost-effectiveness ratio.

The DSAs (Figure 2) revealed the top 5 factors most associated with the ICER, including the OR for healing, the cost of ON101, health utility of healing, probability of transitioning from uninfected to infected DFU, and the cost of hospitalization for an infected DFU. The DSAs indicated that ON101 with GWC vs GWC alone was cost-effective or even cost-saving, except when assuming that the treatment effect size of ON101 with GWC vs GWC alone on healing was 50% lower than the base-case value. The 1-year simulation analysis (eTable 4 in Supplement 1) suggested the short-term cost-effectiveness of ON101 with GWC vs GWC alone (ICER, $9459/QALY gained). The 2-way sensitivity analyses indicated that given a lower monthly ON101 cost of $304, a lower ON101 healing efficacy (OR, ≥1.30) was needed to make the ON101 with GWC strategy cost-effective, but given an increased monthly ON101 cost (up to $912), higher ON101 healing efficacy (OR, ≥1.98) would be required to make the ON101 with GWC strategy cost-effective (eFigure 2 in Supplement 1). The break-even analysis showed that as long as the drug cost of ON101 remained lower than 120% and 190% of the base-case value, ON101 with GWC would still be considered highly cost-effective and cost-effective, respectively, vs GWC alone according to the WTP thresholds of $32 787 to $98 361. If the ON101 cost were lower than 85% of the base-case value, ON101 with GWC could be cost-saving vs GWC alone (eFigure 3 in Supplement 1). Over 10 000 simulations in the PSAs, ON101 with GWC vs GWC alone was associated with a median ICER of $19 891/QALY gained and a mean ICER of −$3917/QALY gained (95% CI, −$261 980/QALY gained to $509 921/QALY gained). The PSAs demonstrated that ON101 with GWC vs GWC alone was considered cost-saving in 29% of the model iterations and cost-effective in 60% and 82% of the model iterations against the WTP thresholds of $32 787 and $98 361, respectively (Figure 3; eFigure 4 in Supplement 1).

### Subgroup Analyses

ON101 with GWC vs GWC alone remained cost-effective across different patient subgroups in a range of 65% to 94% of model iterations against the WTP threshold of $98 361/QALY gained. ON101 with GWC was cost-saving among patients with an HbA_1c_ level of 9% or greater, an ulcer size greater than 5 cm^2^, an ulcer duration of 6 months or longer, or current smoking (eTable 5 in Supplement 1).

## Discussion

Given the results of the ON101 trial,^12^ this economic analysis found that over 5 years, the ON101 with GWC strategy for DFUs was cost-effective from the Taiwan health care sector perspective compared with GWC. This finding was consistent across all sensitivity and subgroups analyses, with 82% of confidence or certainty for the cost-effectiveness of using ON101 against the WTP threshold of $98 361. These economic analysis results are valuable and timely to support the use of ON101 in current practice and encourage the adoption of this breakthrough treatment for DFUs from a health policy perspective.

Healing of DFUs may take months to years, and without complete healing, patients with DFUs are susceptible to a series of costly health consequences associated with deteriorative QoL.^5,27,28^ The randomized clinical trial found a superior therapeutic efficacy of ON101 in the treatment of DFUs in terms of complete healing rate and time to complete healing compared with an absorbent dressing.^12^ Consistently, this 5-year model-based simulation found that a patient using ON101 was not only more likely to reach a healing state more rapidly but also stayed for nearly 6 months longer in the healing state compared with a patient in the GWC group and consequently had a lower risk of progression to DFU-related complications (eTables 2 and 3 in Supplement 1). Taken together, our economic analyses suggest that ON101 with GWC for patients with DFUs was a highly cost-effective treatment strategy compared with GWC.

Given the apparent efficacy on wound healing, it is not surprising to find that the healing efficacy of ON101 with GWC vs GWC alone was the most dominant factor for the economic results in sensitivity analyses. However, this result should be interpreted with caution because we used a wide range for healing efficacy (ie, ±50% of the base-case OR value) to bias against the outcomes associated with ON101, and also, the healing effect size was based on a 28-week clinical trial. The drug cost of ON101 was found to be the second most influential factor for the economic results. ON101 with GWC was still cost-effective even at a 1.9-fold increase in cost (eFigure 3 in Supplement 1), suggesting that the downstream economic benefits following ON101 treatment may in part outweigh its high drug cost. Moreover, owing to the superior healing efficacy of the ON101 treatment, most patients in the ON101 with GWC group progressed to the healing state in the first cycle of the model simulation. Thus, ON101 with GWC would be more cost-effective if the patients perceived a higher value on the humanistic outcome of healing (ie, a greater health utility for the healing state).

Consistent with the findings in the base-case analyses, the ON101 with GWC strategy was cost-effective or even cost-saving across patient subgroups, with 65% to 94% certainty for such cost-effectiveness under the WTP threshold of $98 361 according to the results of PSAs (eTable 5 in Supplement 1). This not only supports the cost-effectiveness of ON101 therapy in diverse DFU populations but also reveals important clinical implications for prioritizing the ON101 with GWC strategy to treat the patients with severe clinical conditions, including those with poor glycemic control (HbA_1c_ ≥9%), an ulcer greater than 5 cm^2^, an ulcer duration of 6 months or longer, or current smoking. Poor glycemic control, large ulcer size, chronic ulcer, and current smoking are known risk factors associated with poor outcomes of DFUs, such as lower-extremity amputation.^29,30,31,32,33^ In fact, approximately one-third of participants in the ON101 trial had a baseline HbA_1c_ level of 9% or greater, ulcer area greater than 5 cm^2^, or ulcer duration of 6 months or longer,^12^ and our analyses would thus support an urgent need for the novel and effective interventions to tackle DFUs and their costly and life-threatening complications among these high-risk patients.

Previous cost-effective studies favor most preventive or treatment strategies for DFUs. A recent systematic review revealed that the intervention with optimal wound care in compliance with clinical guidelines or adjunctive therapies added on to GWC would be cost-effective or cost-saving, except for the use of an epidermal growth factor due to its high acquisition cost.^34^ Among these studies, becaplermin, a biological drug approved by the US Food and Drug Administration for DFUs, was cost-saving as an adjunct to GWC. Unfortunately, due to its potential association with risk of malignant neoplasms,^35^ becaplermin is not commonly used in current practice. Moreover, early intervention of lipido-colloid with nano-oligosaccharide factor dressings vs neutral dressings for DFUs was cost-saving from a French collective payer perspective.^36^ Although caution should be used when directly comparing the results among these studies, our economic analysis is consistent with most previous studies, indicating that an effective intervention to enhance GWC would yield a gain in health benefits at an acceptable or lower incremental cost compared with its counterpart.

### Strengths and Limitations

To our knowledge, this is the first model-based simulation economic analysis of the ON101 with GWC vs GWC alone strategies for DFUs. To ensure the applicability of our economic results to the study setting and the health care sector perspective in the Taiwanese context and to reduce heterogeneity arising from multiple data sources for model input parameters, several methodologic efforts were made, including (1) transformation of the health care costs using the cost ratios to reflect local health care systems and practice patterns; (2) utilization of the Taiwanese population-based health utility data to enhance the applicability of study results to the local setting; (3) adaptation of existing validated disease simulation models to include the gangrene state, which was not well considered in previous cost-effectiveness studies,^15,16,34,36^ given its clinical importance for the Taiwanese patient population with diabetes^37^; (4) implementation of various subgroup analyses to examine the robustness of base-case analysis results and study generalizability to inform clinical practice and health policy decision-making; and (5) implementation of a break-even analysis to inform the health care system of identifying a rational acquisition cost of a new and novel intervention. Also, per guidelines by a model validation-assessment tool, Assessment of the Validation Status of Health-Economic Decision Models,^38^ we made efforts to improve the validation status of the Markov model. They included face validation for the conceptual model, model input data, and model outcomes; cross-validation for the conceptual model and model outcomes; and extreme value testing and 1-way sensitivity analyses to ensure that the model was behaving correctly (debugging).

Some limitations to our study should be acknowledged. First, our analysis may underestimate the cost-effectiveness of ON101 with GWC alone because of 2 conservative modeling assumptions. The relative healing efficacy for ON101 vs GWC alone was adopted directly from the ON101 trial, in which ON101 was compared with a commonly used absorbent dressing for standard care, which has a better wound healing efficacy than GWC.^39^ Therefore, the relative treatment effect size of ON101 with GWC vs GWC alone on complete healing could be greater than that observed in the ON101 trial. Also, health utilities for modeled health states for both groups were assumed to be the same in our analyses because we lacked empirical QoL data. However, from a patient perspective, several practical advantages of ON101 therapy, including self-administration at home after debridement and fewer returning outpatient visits required for wound care, may enhance patients’ treatment compliance and thereby increase their QoL.^40^ As a result, our economic estimates (eg, QALYs) might be underestimated. Second, uncertainty may arise in this 5-year simulation analysis using the healing efficacy data from a clinical trial with only a 28-week follow-up. However, the consistent findings between the sensitivity analysis of a 1-year simulation and the base-case analysis of a 5-year simulation may minimize this concern. Third, some of our efficacy data come from previous studies that were published 20 years ago (Table 1) and may not reflect contemporary clinical settings. However, these parameters were assumed to be the same between the 2 treatment groups, and thus the comparative cost-effectiveness analysis results between groups might be less affected. Also, the cost adaptation and transformation across countries (ie, from Australia to Taiwan) (eFigure 1 in Supplement 1) was made in this study due to the lack of details about individual DFU event costs from our study settings. Despite this effort, one may argue that Australian cost data may be difficult to inform Taiwanese cost settings. With this in mind, a series of sensitivity analyses that varied the values of cost data (Table 1) were performed to examine parameter uncertainty, and the consistent results across the analyses confirm the robustness of our primary findings. Further analysis with up-to-date data comprising the details of treatment efficacy, costs, and health utility penalties associated with more individual DFU events from Taiwan is warranted to confirm our findings. Furthermore, in the estimation of treatment cost, we considered the dosing of ON101 derived from the ON101 trial and did not consider the actual usage or consumption, which would depend on the size and severity of an ulcer.

## Conclusions

Given the high cost-effectiveness of the ON101 with GWC strategy vs GWC alone for treating DFUs from the Taiwan health care sector perspective, the ON101-enhanced GWC may be considered in future standard wound care. Health care payers can consider the implementation of ON101 therapy to patients with an uninfected DFU with a Wagner grade of 1 or 2 and even prioritize it to those with poor glycemic control (HbA_1c_ level, ≥9%), an ulcer size greater than 5 cm^2^, an ulcer duration of 6 months or longer, or current smokers, through reimbursement or low copayments for patients to mitigate the potential access barrier due to a high acquisition cost of ON101. Further research, which comprises more diverse populations with DFUs at different health care settings or from different analysis perspectives, is warranted to assess the external validity of our findings. The evidence on comparative effectiveness and cost-effectiveness of ON101 with GWC therapy would facilitate intervention options to treat DFUs and prevent associated complications.

## Notes

Supplement 1. **eTable 1. **Impact Inventory for Analyses From a Health Care Sector Perspective **eTable 2. **Distribution of the Patient Cohort With DFU-Related Health States Over a 5-Year Model Simulation **eTable 3. **Clinical Effect Sizes of 2 Treatment Groups From 10 000 Microsimulations **eTable 4. **Cost-effectiveness Analysis Results Over a 1-Year Model Simulation **eTable 5. **Subgroup Analyses **eFigure 1. **Two-Stage Cost Transformation Process **eFigure 2. **Two-Way Sensitivity Analyses on the Drug Cost and Healing Efficacy of ON101 Under Predefined WTP Thresholds **eFigure 3. **Break-Even Analysis for the Impact of the ON101’s Drug Cost on the Incremental Cost-Effectiveness Ratio **eFigure 4. **Probabilistic Sensitivity Analysis Results in Incremental Cost-Effectiveness Planes

Supplement 2.
Data Sharing Statement
