# Supplementary material for: Cost-effectiveness of Novel Macrophage-Regulating Treatment for Wound Healing in Patients With Diabetic Foot Ulcers From the Taiwan Health Care Sector Perspective
Source: JAMA Netw Open. 2023 Jan 12;6(1):e2250639. doi: 10.1001/jamanetworkopen.2022.50639 (PMC9856772; doi:10.1001/jamanetworkopen.2022.50639)
Supplement: Supplement 1. — eTable 1. Impact Inventory for Analyses From a Health Care Sector Perspective eTable 2. Distribution of the Patient Cohort With DFU-Related Health States Over a 5-Year Model Simulation eTable 3. Clinical Effect Sizes of 2 Treatment Groups From 10 000 Microsimulations eTable 4. Cost-effectiveness Analysis Results Over a 1-Year Model Simulation eTable 5. Subgroup Analyses eFigure 1. Two-Stage Cost Transformation Process eFigure 2. Two-Way Sensitivity Analyses on the Drug Cost and Healing Efficacy of ON101 Under Predefined WTP Thresholds eFigure 3. Break-Even Analysis for the Impact of the ON101’s Drug Cost on the Incremental Cost-Effectiveness Ratio eFigure 4. Probabilistic Sensitivity Analysis Results in Incremental Cost-Effectiveness Planes [file jamanetwopen-e2250639-s001.pdf]

Supplemental Online Content

Su H, Yang C, Ou H, et al. Cost-effectiveness of novel macrophage-regulating treatment for wound healing in patients with diabetic foot ulcers from the Taiwan health care sector perspective. *JAMA Netw Open.* 2023;6(1):e2250639. doi:10.1001/jamanetworkopen.2022.50639

eTable 1. Impact Inventory for Analyses From a Health Care Sector Perspective

eTable 2. Distribution of the Patient Cohort With DFU-Related Health States Over a 5-Year Model Simulation

eTable 3. Clinical Effect Sizes of 2 Treatment Groups From 10 000 Microsimulations

eTable 4. Cost-effectiveness Analysis Results Over a 1-Year Model Simulation

eTable 5. Subgroup Analyses

eFigure 1. Two-Stage Cost Transformation Process

eFigure 2. Two-Way Sensitivity Analyses on the Drug Cost and Healing Efficacy of ON101 Under Predefined WTP Thresholds

eFigure 3. Break-Even Analysis for the Impact of the ON101's Drug Cost on the Incremental Cost-Effectiveness Ratio

eFigure 4. Probabilistic Sensitivity Analysis Results in Incremental Cost-Effectiveness Planes

This supplemental material has been provided by the authors to give readers additional information about their work.

© 2023 Su H et al. *JAMA Network Open.*

eTable 1. Impact Inventory for Analyses From a Health Care Sector Perspective

Type of impact	Included in this analysis	Notes on sources of evidence
**Health outcome (effects)**		
• Longevity effects	Yes	Published literature
• Health-related quality-of-life effects	Yes	Published literature
• Other health effects (i.e., treatment effect to achieve healing of DFUs and risk of DFUs progression)	Yes	ON101 trial and published literature
**Medical costs**		
• Paid for by third-party payers	Yes	ON101 trial and published literature
• Paid for by patients out-of-pocket	Yes	Published literature
• Future related medical costs (payers and patients)	Yes	Published literature
• Future unrelated medical costs (payers and patients)	No	

Abbreviation: DFUs, diabetic foot ulcers.

© 2023 Su H et al. *JAMA Network Open.*

eTable 2. Distribution of the Patient Cohort With DFU-Related Health States Over a 5-Year Model Simulation

5-year health state distribution	ON101+GWC (%)	GWC (%)
Patients having healing	85.6	79.7
Patients having uDFU	6.5	11.6
Patients having iDFU	1.0	1.3
Patients having post amputation	1.0	1.4

Abbreviations: DFU, diabetic foot ulcer; GWC, general wound care; uDFU, uninfected diabetic foot ulcer; iDFU, infected diabetic foot ulcer.

© 2023 Su H et al. *JAMA Network Open.*

eTable 3. Clinical Effect Sizes of 2 Treatment Groups From 10 000 Microsimulations

Clinical effect	ON101+GWC	GWC	Difference[a]
**Beneficial effect, mean (95% CI)**			
Event of healing	28,852 (28,597-29,107)	26,065 (25,834-26,296)	2,787
Average time spent in healing state (month)	52.6 (52.4-52.8)	47.4 (47.2-47.6)	5.2
**Harmful effect, mean (95% CI)**			
Event of iDFU	6,096 (5,939-6,253)	8,862 (8,664-9,060)	-2,766
Event of amputation	131 (109-153)	203 (174-232)	-72
Event of gangrene	34 (26-42)	41 (29-53)	-7

Abbreviation: CI, confidence interval; GWC, general wound care; iDFU, infected diabetic foot ulcer.

a. GWC was the reference group.

© 2023 Su H et al. *JAMA Network Open.*

eTable 4. Cost-effectiveness Analysis Results Over a 1-Year Model Simulation (Costs in 2021 USD)

Treatment group	Total cost, $	Incremental cost, $	QALY	Incremental QALY	ICER, $ per QALY gained	Probability of cost-effectiveness[a], %
GWC	2,993	---	0.790	---	---	---
ON101+GWC	3,135	143	0.805	0.015	9,459	82

Abbreviations: QALY, quality-adjusted life-year; ICER, incremental cost-effectiveness ratio; GWC, general wound care.

a. The probability was against the willingness-to-pay threshold of $98,361 for a QALY gained.

© 2023 Su H et al. *JAMA Network Open.*

eTable 5. Subgroup Analyses
(Costs in 2021 USD)

Treatment group	Total cost, $	Incremental cost, $	QALY	Incremental QALY	ICER, $ per QALY gained	Probability of cost-effectiveness[b], %
Base-case analysis						
GWC	9,210	---	3.702	---	---	---
ON101+GWC	9,781	571	3.740	0.038	14,922	82
HbA1 <9%						
GWC	9,210	---	0.790	---	---	---
ON101+GWC	9,924	714	3.739	0.037	19,161	79
HbA1c ≥9%						
GWC	9,210	---	3.702	---	---	---
ON101+GWC	9,116	-94	3.745	0.043	-2,187	83
Ulcer size 1-5 cm^2						
GWC	9,210	---	3.702	---	---	---
ON101+GWC	10,411	1,201	3.736	0.034	35,416	65
Ulcer size >5 cm^2						
GWC	9,210	---	3.702	---	---	---
ON101+GWC	8,333	-877	3.750	0.048	-18,153	91
Ulcer duration <6 months						
GWC	9,210	---	3.702	---	---	---
ON101+GWC	9,801	591	3.740	0.038	15,499	74
Ulcer duration ≥6 months						
GWC	9,210	---	3.702	---	---	---
ON101+GWC	8,234	-976	3.751	0.049	-19,922	94
Non-current smoker[a]						
GWC	9,210	---	3.702	---	---	---
ON101+GWC	10,335	1,126	3.736	0.034	32,692	71
Current smoker						
GWC	9,210	---	3.702	---	---	---
ON101+GWC	8,323	-886	3.750	0.048	-18,321	93

Abbreviations: QALY, quality-adjusted life-year; ICER, incremental cost-effectiveness ratio; GWC, general wound care.

a. Non-current smoker included non-smoker and former smoker.

b. The probability was against the willingness-to-pay threshold of $98,361 for a QALY gained.

© 2023 Su H et al. *JAMA Network Open.*

eFigure 1. Two-Stage Cost Transformation Process

(a)

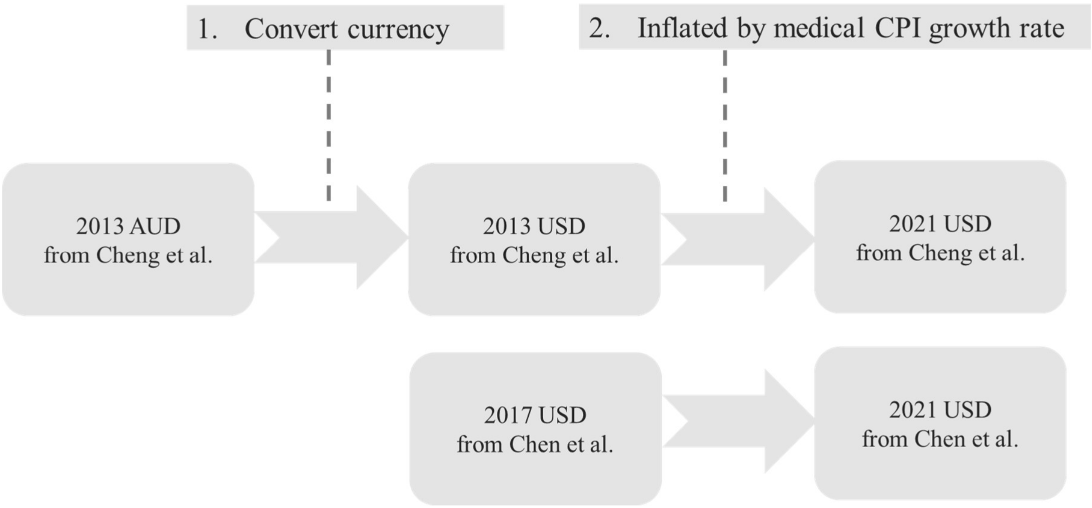

(b)

Cost parameters	Cheng et al. (in 2021 USD)	Chen et al. (in 2021 USD)
Hospitalization for amputation	14,550	Ratio 1 → 7,552
Hospitalization for infection	17,045	→
Hospitalization for post amputation infection	26,169	→
Hospitalization for post amputation gangrene	26,169	→
Initial DFU	309	→
Primary healed	48	→
Uninfected DFU	526	→
Infected DFU	865	→
Gangrene	1,250	→
Post amputation	2,727	Ratio 2 → 504

Abbreviations: CPI, consumer price index; AUD, Australia dollar; USD, United States dollar; DFU, diabetic foot ulcer.

(a) Costs from two studies (i.e., Cheng et al. in Australia and Chen et al. in Taiwan) were converted into the same currency (i.e., USD) and standardized into year 2021 values using Taiwan's medical CPI; (b) Cost ratios for transformation were calculated by dividing the Taiwan's cost estimates by the Australian estimates for the health states which were available in both Taiwan's and Australian data. Ratio 1 represented the ratio for transformation of the event costs (e.g., hospitalization for amputation) from Australian data into Taiwan's data. Ratio 2 represented the ratio for transformation of the state costs (e.g., post amputation) from Australian data into Taiwan's data. Then, we applied these two cost ratios to transform medical costs of other DFU-related health states provided by Cheng et al.'s study as cost inputs for our economic analysis.

© 2023 Su H et al. *JAMA Network Open.*

eFigure 2. Two-Way Sensitivity Analyses on the Drug Cost and Healing Efficacy of ON101 Under Predefined WTP Thresholds

(a)

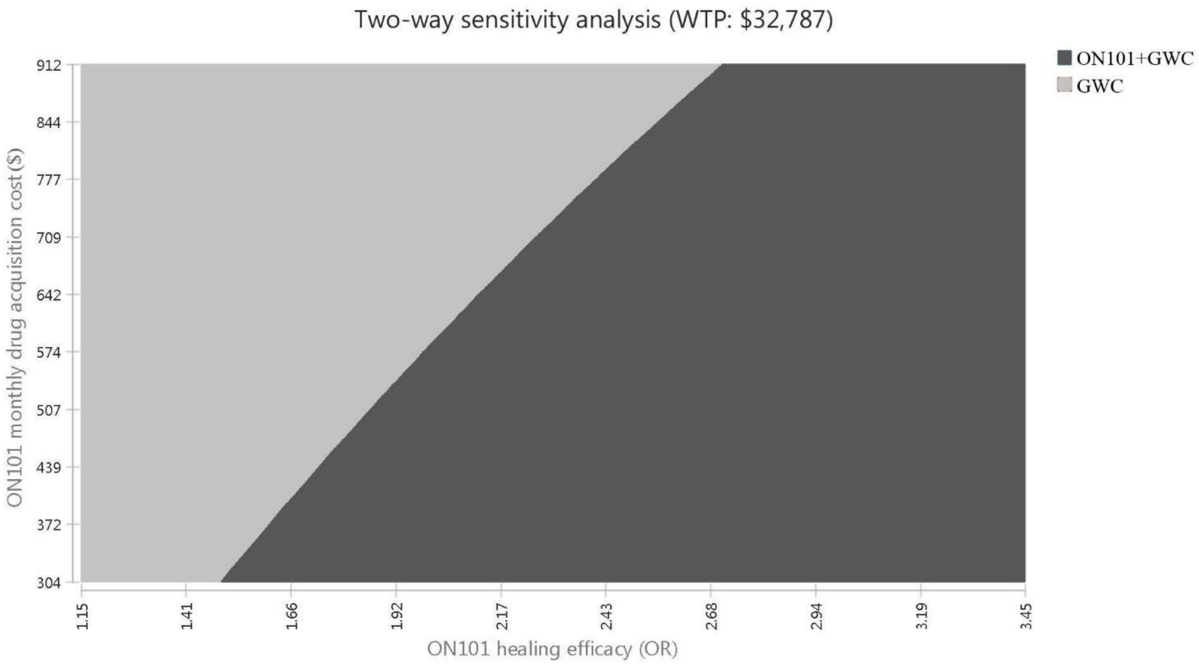

(b)

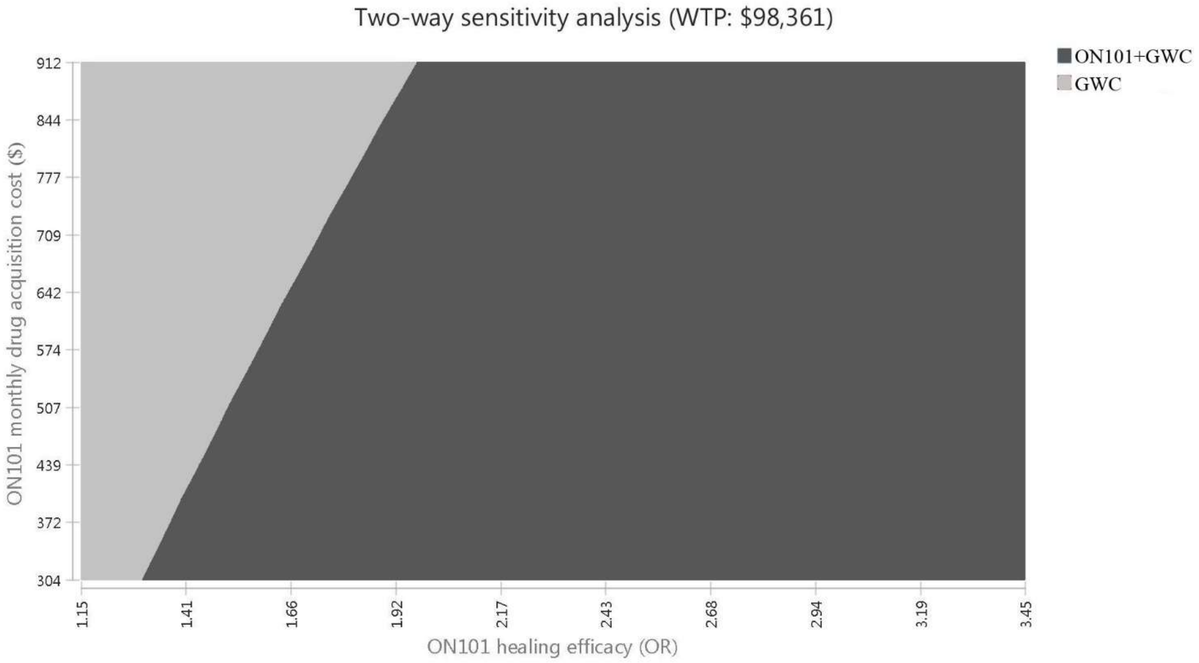

Abbreviations: OR, odds ratio; GDP, gross domestic product; WTP, willingness-to-pay; USD, United States dollar; GWC, general wound care.

Legend: (a) using one-time GDP per Taiwan's capita ($32,787) as the WTP, (b) using three-time GDP per Taiwan's capita ($98,361) as the WTP. As the cost (presented as percentage of base-case value) and healing efficacy (i.e., OR) of ON101 change across a range of values, the comparative cost-effectiveness between ON101 added-on to GWC (i.e., ON101+GWC) and GWC also changes. In this graph, two-dimensional area based on the simultaneous change of the cost and healing efficacy of ON101 is displayed to determine an optimal treatment. Specifically, the gray area

© 2023 Su H et al. *JAMA Network Open.*

corresponds to model input values that will result in the GWC as more cost-effective, while the black area corresponds to model input values that will result in the ON101+GWC as more cost-effective. Based on this graph, the ON101+GWC strategy can be considered as an optimal treatment, regardless of the pre-defined WTP thresholds.

© 2023 Su H et al. *JAMA Network Open.*

eFigure 3. Break-Even Analysis for the Impact of the ON101's Drug Cost on the Incremental Cost-Effectiveness Ratio

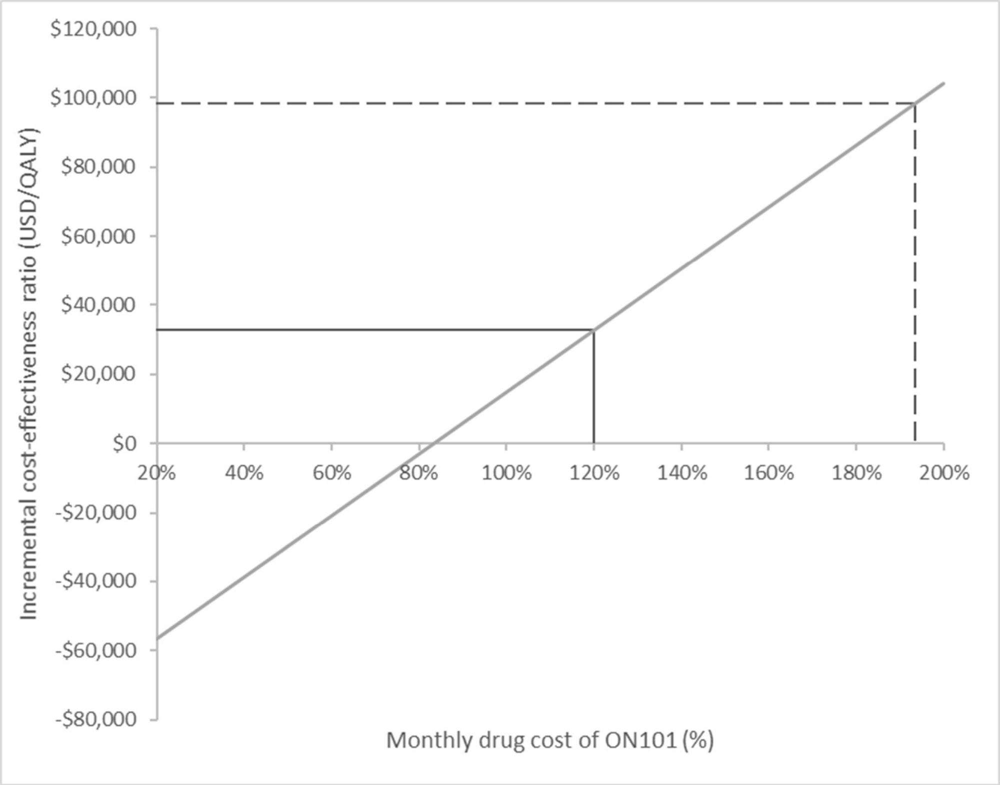

Abbreviations: QALY, quality-adjusted life-year; WTP, willingness-to-pay.

X-axis indicates the percent of monthly drug cost of ON101 increase against the base-case value and Y-axis shows the corresponding incremental cost-effectiveness ratios of ON101 added to GWC versus GWC. The black solid line indicates when the WTP is one time per Taiwan's capita gross domestic product (GDP) (i.e., $32,787), the break-even drug acquisition cost of ON101 is about 120% of base-case value; and black dash line indicates when the WTP is 3 times per country's capita GDP (i.e., $98,361), the break-even drug cost of ON101 is about 194% of base-case value.

© 2023 Su H et al. *JAMA Network Open.*

eFigure 4. Probabilistic Sensitivity Analysis Results in Incremental Cost-Effectiveness Planes

(a)

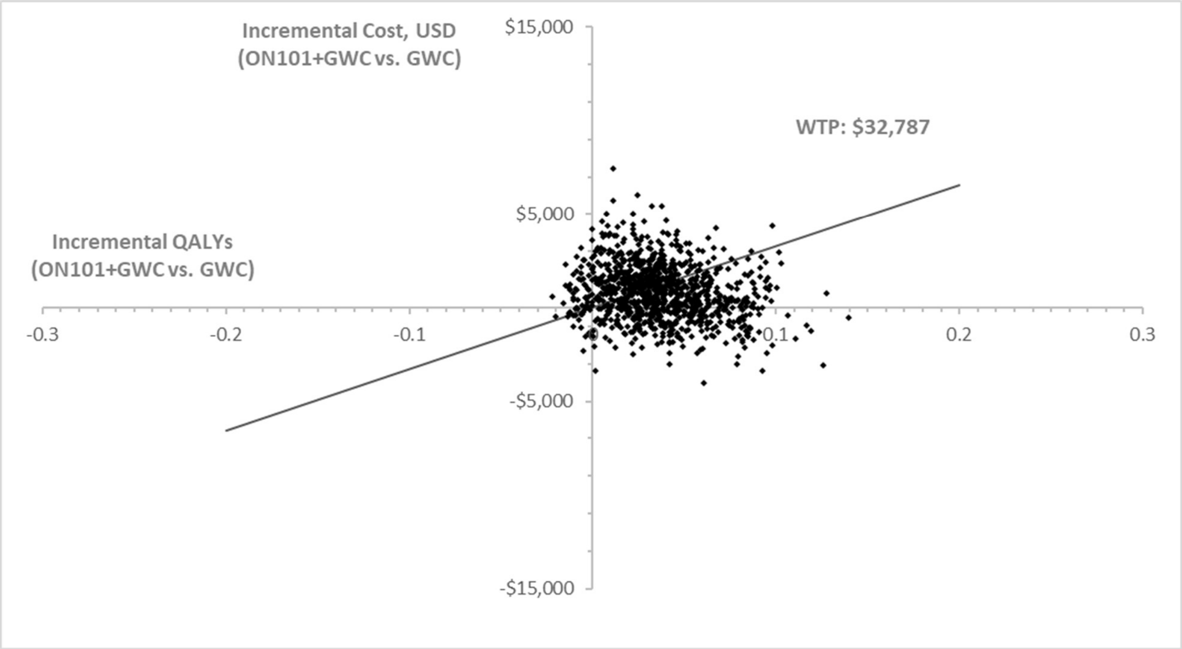

(b)

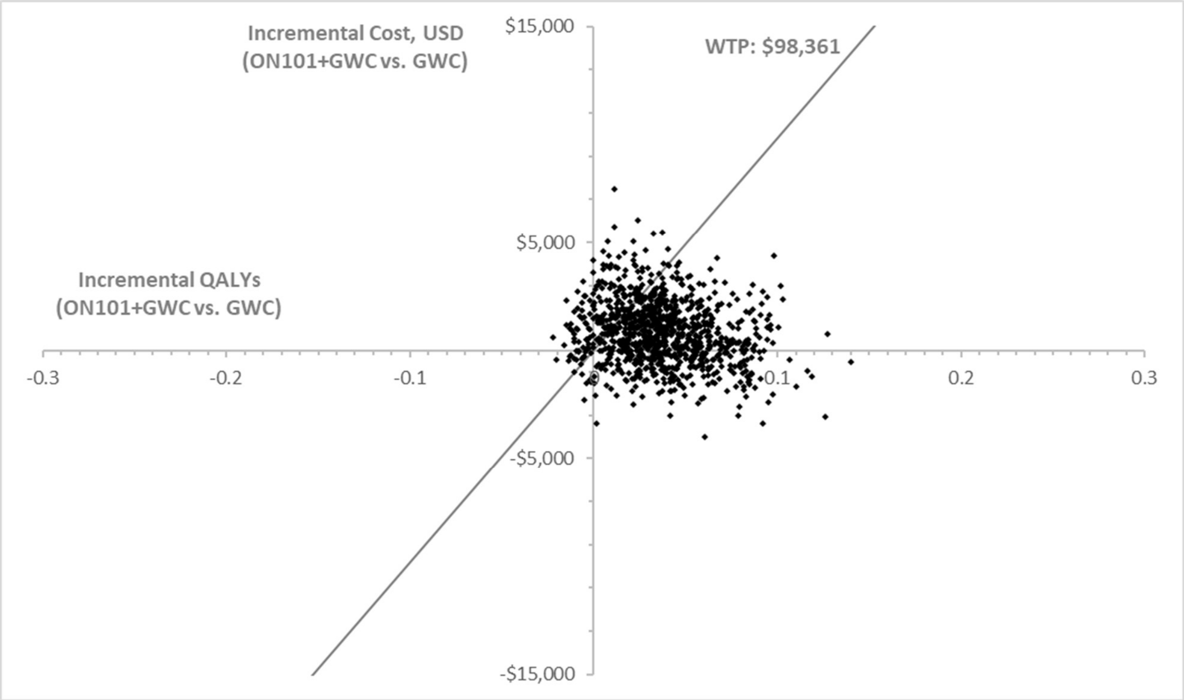

The figures show the probabilistic uncertainty of cos-effectiveness of ON101 added on to GWC (ON101+GWC) versus GWC based on the base-case analysis with 10,000 model iterations. The x-axis represents the incremental quality-adjusted life-years (QALYs); the y-axis represents the incremental cost. In figure (a), the solid line represents a willingness-to-pay (WTP) threshold of $38,727 per QALY gained, corresponding to one time the per capita gross domestic product (GDP) in Taiwan, resulting in 59.7% probability of using ON101+GWC being cost-effective; in figure (b) the solid line represents a WTP threshold of $98,361 per QALY gained, three times the capita GDP in Taiwan, resulting in 81.8% model iterations for using ON101+GWC being cost-effective.

© 2023 Su H et al. *JAMA Network Open.*
