# Supplementary material for: Cost-effectiveness of Novel Macrophage-Regulating Treatment for Wound Healing in Patients With Diabetic Foot Ulcers From the Taiwan Health Care Sector Perspective
Source: JAMA Netw Open. 2023 Jan 12;6(1):e2250639. doi: 10.1001/jamanetworkopen.2022.50639 (PMC9856772; doi:10.1001/jamanetworkopen.2022.50639)
Supplement: Supplement 2. — Data Sharing Statement [file jamanetwopen-e2250639-s002.pdf]

Data Sharing Statement

Su. Cost-effectiveness of Novel Macrophage-Regulating Treatment for Wound Healing in Patients With Diabetic Foot Ulcers From the Taiwan Health Care Sector Perspective. *JAMA Netw Open*. Published January 12, 2023. doi:10.1001/jamanetworkopen.2022.50639

Data
Data available: No
